# Risk Factors for post-Cardiac Surgery Diaphragmatic Paralysis in Children with Congenital Heart Disease 

**Published:** 2015-07-03

**Authors:** Parvin Akbariasbagh, Mohammad Reza Mirzaghayan, Naseredin Akbariasbagh, Mamak Shariat, Bita Ebrahim

**Affiliations:** 1 *Imam Khomeini Complex, Tehran University of Medical Sciences, Tehran, Iran. *; 2 *Baharlo Hospital, Tehran University of Medical Sciences, Tehran, Iran.*; 3 *Maternal-Fetal and Neonatal Research Center, Tehran University of Medical Sciences, Tehran, Iran. *; 4 *Valiasr Hospital, Breastfeeding Research Center, Tehran, Iran.*

**Keywords:** *Cardiac surgical procedures*, *Phrenic nerve*, *Diaphragmatic*, *Infant*, *newborn*

## Abstract

**Background:** Injured phrenic nerve secondary to cardiac surgeries is the most common cause of diaphragmatic paralysis (DP) in infants. The aim of this study was to determine the risk factors for DP caused by congenital heart defect corrective surgeries in pediatrics.

**Methods:** This cross-sectional study, conducted in a 2-year period (2006–2008), included 451 children with congenital heart diseases admitted to the Pediatric Cardiac Surgery Ward of Imam Khomeini Hospital. The diaphragmatic function was examined via fluoroscopy, and the frequency of DP and its relevant parameters were evaluated.

**Results**: Of the 451 patients, comprising 268 males and 183 females at an age range of 3 days to 204 months (28.2 ± 33.4 months), 25 (5.5%) infants (60% male and 40% female, age range = 15 days to 132 months, 41.2 ± 28.1 months) had DP as follows: 48% unilateral right-sided and 36% unilateral left-sided. Additionally, 68% had cyanotic congenital heart disease and 84% had DP following total correction surgery. The highest prevalence rates of DP resulting in phrenic hemiparesis were observed after arterial switch operation, Fontan procedure, and Blalock–Taussig shunt surgery, respectively. Thirteen (52%) of the 25 DP patients underwent surgical diaphragmatic plication because of severe respiratory distress and dependency on mechanical ventilation, and most of the cases of plication underwent arterial switch operation. The rate of mortality was 24% (6 patients).

**Conclusion:** DP with a prevalence of 5.5% was one of the most common complications secondary to cardiac surgeries in the infants included in the present study. Effective factors were age, weight, cyanotic congenital heart defects, and previous cardiac surgery. Diaphragmatic plication improved prognosis in severe cases.

## Introduction

Congenital heart diseases are seen in 0.8% of live births.^1^ In their first year of life, 2-3 neonates per 1000 are diagnosed with heart disease: 50%-60% of them are diagnosed in their first month and 40%-50% in their first week of life. The etiology of most congenital heart defects is unknown inasmuch as these diseases are often multi-factorial and combinations of genetic and environmental factors play a role in their incidence.^1^

Cardiac surgeries at a young age could pose high risks and cause complications such as diaphragmatic paralysis (DP).^2^ In fact, nowadays injured phrenic nerve secondary to cardiac surgeries is the most common cause of DP in infants (prevalence = 0.3-12.8%).^3^ It can be induced by amputation, crushing, stretching, or thermal nerve injury (hypothermia caused by contact with cold saline or hyperthermia due to contact with the cautery plate during surgery).^3^^, ^^4^

In infants and neonates, the compliance of the chest wall and mobility of the mediastinum are more than those in adults. Therefore, when diagnosed with DP, neonates and infants present more clinical symptoms than older children and adults.^3^ Usually, poor weaning is the first sign of DP. Indeed, recurrent respiratory distress, atelectasis, and recurrent pneumonia after extubation can be the other signs of DP.^3^^, ^^5^

The phrenic nerve dysfunction leads to the paradoxical movement of the diaphragm during inspiration and expiration, preventing not only the expansion of the collapsed lower lobe of the involved lung but also the efficient inflation of the upper lobe of the same lung.^6^

DP is strongly suggested when a patient has respiratory distress after being weaned from the ventilator and needs reintubation. An elevated hemidiaphragm on chest X-ray is suggestive of DP, but definite diagnosis will be confirmed with fluoroscopy during spontaneous breathing.^5^ In a study conducted by Sanchez de Toledo J. et al.,^7^ ultrasound with a high diagnostic value was used as an easy and valid clinical test to examine the diaphragmatic movement. Also, it should be noted that temporary DP is observed in some cases after surgery. A waiting period of at least one week and a record review of the patient are highly recommended before opting for plication surgery.^8^

## Methods

In this descriptive cross-sectional study, 470 children with congenital heart defects admitted to the Pediatric Cardiac Surgery Ward of Imam Khomeini Hospital between 2006 and 2008 were studied for the prevalence of postoperative DP and its probable risk factors. Nineteen patients were excluded from the study because they died during or shortly after surgery due to other cardiac surgery complications such as cardiac arrhythmia, cardiac and respiratory arrest, bleeding, and electrolyte disorder. Hence, the study was performed on the remaining 451 patients. 

All the patients were transferred to the Intensive Care Unit for postoperative care, and they all underwent mechanical ventilation. First, the ventilator was set on the volume-control mode. As soon as the patient was able to breathe spontaneously and ready to be weaned, the ventilator was set on the synchronized intermittent mandatory ventilation mode. The hemodynamically stable patients with sufficient levels of consciousness to maintain airway protective reflexes (coughing and gagging) and adequate oxygenation to be confirmed via the arterial blood gas analysis were weaned usually during the first 2 days after surgery. Post-surgery phrenic nerve palsy was suggested in cases who were not able to be weaned or had respiratory distress after weaning, requiring reintubation. In these patients, breath sounds were decreased, and the chest and abdominal movements were paradoxical. Chest X-ray was performed on the patients with the aforementioned postoperative symptoms: the results were strongly suggestive of DP, especially in the cases with the unilateral elevation of the diaphragm. Fluoroscopy was performed in order to confirm the diagnosis. Following diagnosis, the patients less than 6 kg and under 1 year of age, and the ones who were not able to be weaned from the ventilator or those who had developed severe respiratory distress after extubation underwent diaphragm repair through plication surgery. In the other cases, the main treatment method was supportive care, including respiratory physiotherapy.

The frequency of DP and its possible related variables such as age, gender, and weight as well as the type of heart disease (cyanotic or non-cyanotic), type of surgery (total correction or palliative), presence or absence of previous cardiac surgery, surgical approach, type of diaphragmatic injury, duration of hospital stay, cases of mortality, and treatment procedures for DP were extracted from the patients’ records available in the medical archives of Imam Khomeini Hospital. All the above-mentioned information was obtained via a careful examination of data files, including biographies, physician order sheets, practice sheets, and daily notes documented by the physician. Also, the three separate daily nursing reports (morning, afternoon, and night) of the patients’ condition at the Intensive Care Unit had an important role in the weaning process. The required data were collected from the patients’ medical records without their names; the information included their file numbers recorded in completely confidential checklists. The DP patients were contacted using their phone number and home address registered in their medical records during their hospital stay for a follow-up appointment. Despite some changes in addresses listed in their files, the patients were asked to refer to the hospital for fluoroscopy. Fluoroscopy was performed by a radiologist in order to evaluate the diaphragmatic movements, the results of which were presented in two categories: 1) motionless diaphragm or paradoxical movements during respiration and 2) normal bilateral movements.

For the statistical analyses, the Statistical Package for the Social Sciences (SPSS), version 16.0, for Windows (SPSS Inc., Chicago, IL) was used. The quantitative variables with a normal distribution were examined via the t-test, and the ones without a normal distribution were assessed via the Mann-Whitney U test. The chi-squared test, Fisher exact test, and, logistic regression model were used for the qualitative variables. Also, the One-Way Analysis of Variance (ANOVA) and post-hoc Tukey test were employed to compare the quantitative variables between the groups, and a p value of 0.05 was considered significant.

The parents of the patients gave informed consent before any surgery, and they were already aware of the risks and possible post-surgical complications such as DP. In the event of DP, the parents were informed about their patient’s condition and subsequent complications induced by the disease, as well as the necessary treatment measures in order to repair the diaphragm and necessity of regular medical follow-up and referrals. The present study was approved by the Ethics Committee of Tehran University of Medical Sciences.

## Results

The prevalence of the postoperative DP was retrospectively studied in a total of 451 children diagnosed with congenital heart defects. Of the 451 patients, 25 (5.5%) developed DP following surgery. The data on the demographic variables, types of congenital cardiac disease, types of surgery, sternotomy approaches, and previous history of cardiac surgery are shown in Table 1.

Significant differences were seen between the groups in terms of age (odds ratio [OR] = 0.005; p value = 0.025) and weight (OR = 0.042; p value < 0.001). Hospital stay in the patients diagnosed with DP was significantly more prolonged than that in the non-DP patients (OR = 0.14; p value = 0.0001). There was no statistically significant difference between the two groups in terms of gender (OR = 1; p value = 0.100). In contrast, a statistically significant difference was seen between cyanotic and non-cyanotic congenital heart defects in the two groups, and the prevalence of DP was more common in the children with cyanotic congenital heart disease (OR = 0.64; p value = 0.048). The rate of mortality in the DP patients was 25.0% (6 patients) compared to 5.4% in the non-DP patients (OR=3.33; p value = 0.031, [95%CI: 1.136-13.647]). Evidently, there was a significant difference in mortality between the two groups in that the rate of mortality was higher in the patients diagnosed with DP.

Most cases of DP were caused by the Fontan procedure, Blalock–Taussig shunt surgery, and arterial switch operation. The prevalence rate of the post-surgical injuries in all the three surgeries was 16.6%. No statistically significant difference was seen between the two groups in terms of the type of surgery (total correction or palliative) (OR = 0.07; p value = 0.999). 

Statistically, there was no significant difference in the surgical approaches between the two groups, but DP was more common in the patients with a previous history of surgery (OR = 6; p value = 0.002).

**Table 1:** Demographic characteristics*

	Children Diagnosed with Diaphragmatic Paralysis (n=25)	Children without Diaphragmatic Paralysis (n=426)
Males	15 (60)	253 (59.4)
Age (mo)	41.2±28.1	28.2±33.4
Weight (kg)	7.1±2.7	11.4±8.1
Hospital stay duration (d)	31.2±28.6	11.2±6.9
Cyanotic congenital cardiac diseases	17 (68.0)	180 (42.3)
Non-cyanotic congenital cardiac diseases	8 (32.0)	246 (57.7)
Type of Surgery		
Total correction surgery	21 (84.0)	350 (82.2)
Palliative surgery	4 (16.0)	76 (17.8)
Type of sternotomy		
Median sternotomy	23 (92.0)	389 (91.3)
Posterolateral thoracotomy	2 (8.0)	37 (8.7)
Previous cardiac surgery history		
Performed	14 (56.0)	72 (16.9)
Not performed	11 (44.0)	354 (83.1)
Type of paralysis		
Unilateral right-sided	12 (48.0)	0
Unilateral left-sided	9 (36.0)	0
Bilateral	4 (12.0)	0
Mortality	6 (24.0)	23 (5.4)

* Data are presented as mean±SD or n (%).

**Table 2:** Related variables based on logistic regression model

Variable	B	OR	95%CI	P Value
Age	-0.004	0.005	0.008-0.001	0.025
Gender	0.000	1.000	0.361-2.773	1.000
Weight	0.052	0.042	0.035-0.069	< 0.001
Cyanotic congenital cardiac diseases	-1.056	0.640	0.122-0.992	0.048
Previous cardiac surgery	1.792	6.000	1.928-18.672	0.002
Type of surgery	-21.801	0.070	0.003-1.250	0.999
Duration of hospital stay	-0.017	0.140	0.220-0.011	< 0.001
Mortality	-1.371	3.330	0.073-0.880	0.031

Of the 25 patients diagnosed with DP, 12 (48%) had unilateral right-sided, 9 (36%) unilateral left-sided, and 4 (16%) bilateral DP. Despite plication, 6 (24%) patients underwent tracheostomy due to severe respiratory distress and difficulty in being weaned from the ventilator. Of these 6 patients, 4 were diagnosed with bilateral, 1 with unilateral right-sided, and 1 with unilateral left-sided DP. Of the 25 DP patients, 13 (52%) underwent diaphragm plication surgery and 12 (48%) received supportive care. The frequency of the types of surgery leading to DP and required plication is as follows:

One case of valvotomy, 1 case of Blalock–Taussig shunt surgery, 1 case of Glenn surgery, 2 cases of tetralogy of Fallot correction surgery, 3 cases of ventricular septal defect closure, 1 case of coarctation repair, and 4 cases of arterial switch surgery. It can, therefore, be concluded that most of the plication cases were secondary to arterial switch surgery. The related variables are depicted in Table 2.

Based on our logistic regression model, the effective factors were age, weight, cyanotic congenital heart defects, and previous history of cardiac surgery.

Of the 25 DP patients, 6 cases died. Of the remaining 19 DP patients, 13 cases underwent fluoroscopy to examine their diaphragmatic movements. Six patients were out of reach because of address change. Of the 13 patients, 5 (38.5%) had plication surgery following DP, and the mean time from plication to follow-up fluoroscopy was 18 to 63 months (41.8 ± 6.8). Of these 13 patients, 11 (84.6%) had normal and symmetrical diaphragmatic movement on both sides. In 2 (15.4%) patients, the paralyzed side showed abnormal movement. Paradoxical movement during respiration was not reported. Both patients had a history of unilateral left-sided DP; however, one of them received supportive care and the other one had a history of plication. Both had total correction surgery, leading to the phrenic nerve palsy. Median sternotomy was performed in one case and left posterolateral thoracotomy in the other one. The 11 patients with normal and symmetrical movement of the diaphragm comprised 6 patients with a history of unilateral right-sided DP, 4 unilateral left-sided DP, and 1 bilateral paralysis. Four (36.3%) of these patients underwent plication surgery and 7 (63.6%) received supportive care. 

## Discussion

The phrenic nerve damage is one of the recognized complications of cardiac surgeries leading to DP and respiratory dysfunction. Most cases of the phrenic nerve damage over the last few years have been caused by trauma during delivery. However, the phrenic nerve damage during surgery has been the most common cause of DP in children in recent years, which is due to the increasing number of cardiac surgeries.^5^^ , ^^9^

The present study examined the prevalence of the phrenic nerve disorder and its relevant variables. Of 451 patients who underwent cardiac surgery, 25 developed postoperative DP with a prevalence of 5.5%, which is comparable to other similar studies. The prevalence of this complication was 4.1% in the Lemmer et al.^4^ study, 5.4% in the Joho-Arreola et al.^5^ study, 10% in the Mok et al.^9^ study, 4.9% in the Akay et al.^10^ study, 1.6% in the De Leeuw et al.^11^ study, and 1.9% in the Van Onna et al.^12^ study.

Indeed, dissimilar rates of DP have been reported in different countries. It seems that this discrepancy is in consequence of different study methods rather than different surgical techniques. For instance, Mok et al.^9^ reported the highest incidence of DP (10%) in London. This can be attributable to a new method in the diagnosis of the phrenic nerve damage by the percutaneous stimulation of the phrenic nerve, which is much more sensitive than the common diagnostic methods.

In the present study, the mean age of the patients diagnosed with DP was 41.2 ± 28.1 months, as opposed to that in other studies ranging from 66 days to 15 months.^3^^, ^^5^^, ^^9^^-^^12^ In the studies performed by Baker et al.^3^ and Van Onna et al.,^12^ similar to our study, the disease was more prevalent in boys. 

Of our 25 DP patients, 68% suffered from cyanotic congenital heart disease and 32% had a history of non-cyanotic congenital heart disease. DP occurred in 84% of the patients secondary to total correction surgery, and only in 16% was it induced by palliative surgeries. The highest rate of prevalence was observed after Blalock–Taussig shunt surgery, Fontan procedure, and arterial switch operation (16.6% each). In the Akay et al.^10^ study, the highest incidence rates of postoperative PD were after tetralogy of Fallot corrective surgery, Blalock–Taussig shunt operation, ventricular septal defect closure surgery, and pulmonary artery patch plasty. Similarly, in a study conducted by Joho-Arreola et al.,^5^ the most common causes were Blalock–Taussig shunt surgery, Fontan procedure, and arterial switch operation. The above results are due to the fact that congenital heart defect corrective surgeries require open heart surgery; consequently, more structural changes are made to repair the present defect than in palliative surgeries, increasing the risk of the phrenic nerve damage secondary to these surgeries. 

In the present study, right-sided DP was the most common defect, followed by left-sided and bilateral diaphragmatic damage. Right-sided DP was the most common in our study and that by Simansky et al.,^13^ whereas in the studies conducted by Baker et al.,^3^ Lemmer et al.,^4^ Mok et al.,^9^ and Van Onna et al.,^12^ left-sided paralysis was the most prevalent. In the current study, as well as in the studies conducted by Lemmer et al.,^4^ Mok et al.,^9^ and Simansky et al.,^13^ the presence of a significant association between the history of cardiothoracic surgery and the phrenic nerve damage has been confirmed.

According to the results of the present study and other similar studies mentioned above, a previous history of cardiothoracic surgery with fibrosis and adhesions in the site of operation creates some difficulties in finding the phrenic nerve during surgery. Accordingly, the probability of unintended injuries, including thermal injury, contusion, rupture, and nerve tension, rises during surgery. Also, the risk of the phrenic nerve damage increases during repeated surgeries, and it is often due to the adhesion of the lungs to the heart chambers, followed by dissection made in order to separate the lungs from the heart.

In a study conducted by Mok et al.,^9^ 6 of the 10 patients underwent median sternotomy. Chiming in with our results, the authors reported that the phrenic nerve damage secondary to cardiac surgeries via lateral thoracotomy was not more prevalent than that in the other patients who went through the median sternotomy procedure. Due to the anatomical path of the phrenic nerve, lateral thoracotomy does not seem to be leading to DP and phrenic nerve damage more than the median sternotomy procedure.

In the present study, 52% of the DP patients with severe respiratory distress and poor weaning underwent placation surgery. The DP patients who were younger and weighed less than the others were in more serious need for plication surgery owing to the respiratory dysfunction induced by DP. Since the arterial switch operation is generally performed during early infancy, patients diagnosed with the phrenic nerve palsy subsequent to surgery are in more need for plication.

In a study conducted by Baker et al.,^3^ the rates of the phrenic nerve damage secondary to the Glenn operation, Norwood surgery, and Fontan procedure requiring plication were reported at 3.5%, 2.9%, and 0.75%, correspondingly. Based on the Simansky et al.^13^ study, all of the children diagnosed with DP underwent plication surgery in order to be weaned from the ventilator and breathe spontaneously, while fewer than 50% of the adults needed diaphragm plication surgery. In other studies, patients requiring plication surgery were younger than the group that received supportive therapy.^3^^-^^5^^, ^^9^^-^^13^ The results of the current study and the aforementioned studies confirm that the risk of developing severe respiratory complications subsequent to DP is higher during early infancy. This complication could be the result of undeveloped intercostal muscles in this age group. Accordingly, spontaneous breathing completely depends on the diaphragm; diaphragm plication surgery is necessary to improve symptoms and extubate the patient successfully.

In the present study, the mean duration of hospital stay in the DP patients, especially in those with bilateral DP and the ones who underwent plication surgery subsequent to DP, was longer than that in the patients treated via supportive therapy measures. The results of the De Leeuw.^11^ study are similar to ours.

Despite plication surgery, 6 (24%) patients due to respiratory distress and ventilator dependency underwent tracheostomy. Of the 46 DP patients examined in the study by Baker et al.,^3^ tracheostomy tube placement proved necessary in 3 due to severe respiratory distress despite plication. Also in the Lemmer et al.^4^ study, 5 of the 47 patients with DP required tracheostomy: bilateral DP was observed in 2 of these patients. 

The rate of mortality induced by DP was 24% in the present study, 19.1% in the Akay et al.^10^ study, 23.2% in the Joho-Arreola et al.^5^ study, and 40% in the Mok et al.^9^ study.

Our patients underwent fluoroscopy with monthly follow-up: 84.6% had symmetric and normal movements on both sides of the diaphragm. In the Baker et al.^3^ study, 17 of the 46 patients with DP underwent fluoroscopic control after plication from 2.4 to 89.7 months (16.4 months on average): in all the cases except for one, the return of the plicated diaphragm to its normal function was observed. In the study by Van Onna et al.,^12^ the average fluoroscopic follow-up of patients with DP was 19 ± 5 months after placation: 41% of these patients had plicated diaphragms with a normal function. 

The major limitation of our study was the loss to follow-up of some patients. A more meticulously devised discharge protocol is required to address this shortcoming. 

## Conclusion

The results of the present study showed that DP with a prevalence of 5.5% was one of the most common complications secondary to cardiac surgeries. The rate of mortality was 24%, which was significantly high in comparison with 5.4% mortality in the patients who underwent surgery without complications. DP led to a considerably prolonged hospital stay, giving rise to various complications and a significant increase in morbidity. Our results revealed that most cases of DP occurred secondary to total correction surgeries such as arterial switch operation and in the majority of the patients with cyanotic heart disease who had a history of at least one previous cardiothoracic surgery. The incidence of DP, however, was not associated with the surgical approaches. Other similar studies have reported no correlation between DP and surgical technique, surgeon's experience, and surgical approach.

## Notes:

***This paper should be cited as:*** Akbariasbagh P, Mirzaghayan MR, Akbariasbagh N, Shariat M, Ebrahim B. Risk Factors of post-Cardiac Surgery Diaphragmatic Paralysis in Children with Congenital Heart Disease. J Teh Univ Heart Ctr 2015;10(3):134-139.
